# Role of gamma carboxylated Glu47 in connexin 26 hemichannel regulation by extracellular Ca^2+^: Insight from a local quantum chemistry study

**DOI:** 10.1016/j.bbrc.2014.01.063

**Published:** 2014-02-28

**Authors:** Francesco Zonta, Fabio Mammano, Mauro Torsello, Nicola Fortunati, Laura Orian, Antonino Polimeno

**Affiliations:** a Dipartimento di Fisica e Astronomia “G. Galilei”, Università degli Studi di Padova, 35131 Padova, Italy; b Istituto Veneto di Medicina Molecolare, Fondazione per la Ricerca Biomedica Avanzata, 35129 Padova, Italy; c Istituto CNR di Neuroscienze, 35131 Padova, Italy; d Dipartimento di Scienze Chimiche, Università degli Studi di Padova, Via Marzolo 1, 35131 Padova, Italy

**Keywords:** Hybrid DFT calculations, Calcium ions, Connexin mutations, Gating, Deafness, Charcot Marie Tooth disease

## Abstract

Connexin hemichannels are regulated by several gating mechanisms, some of which depend critically on the extracellular Ca^2+^ concentration ([Ca^2+^]_e_). It is well established that hemichannel activity is inhibited at normal (∼1 mM) [Ca^2+^]_e_, whereas lowering [Ca^2+^]_e_ to micromolar levels fosters hemichannel opening. Atomic force microscopy imaging shows significant and reversible changes of pore diameter at the extracellular mouth of Cx26 hemichannels exposed to different [Ca^2+^]_e_, however, the underlying molecular mechanisms are not fully elucidated. Analysis of the crystal structure of connexin 26 (Cx26) gap junction channels, corroborated by molecular dynamics (MD) simulations, suggests that several negatively charged amino acids create a favorable environment for low-affinity Ca^2+^ binding within the extracellular vestibule of the Cx26 hemichannel. In particular a highly conserved glutammic acid, found in position 47 in most connexins, is thought to undergo post translational gamma carboxylation (γGlu47), and is thus likely to play an important role in Ca^2+^ coordination. γGlu47 may also form salt bridges with two conserved arginines (Arg75 and Arg184 in Cx26), which are considered important in stabilizing the structure of the extracellular region.

Using a combination of quantum chemistry methods, we analyzed the interaction between γGlu47, Arg75 and Arg184 in a Cx26 hemichannel model both in the absence and in the presence of Ca^2+^. We show that Ca^2+^ imparts significant local structural changes and speculate that these modifications may alter the structure of the extracellular loops in Cx26, and may thus account for the mechanism of hemichannel closure in the presence of mM [Ca^2+^]_e_.

## Highlights

• QM calculations show that Ca^2+^ binds to γGlu47 in connexin hemichannels.
• Molecular models of increasing size are employed in hybrid DFT calculations.
• Ca^2+^ binding affects the interaction between γGlu47 and Arg75, Arg184.
• Ca^2+^ binding alters the structure in a critical region of connexin hemichannels.

## Graphical abstract

**Figure:**
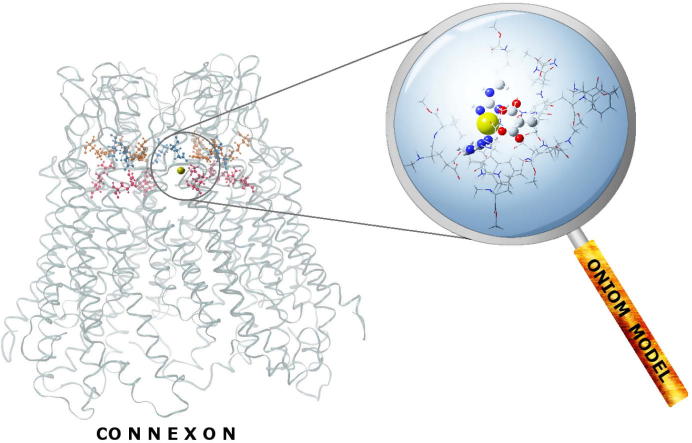

## 1 Introduction

Connexins, indicated with the abbreviation Cx followed by their molecular weight in kilodalton (e.g. Cx26 for the 26 kDa protomer), are tetraspan transmembrane proteins encoded by at least 21 genes in the human and 20 genes in the mouse genome, 19 of which can be grouped as sequence-orthologous pairs [1]. Connexin proteins oligomerize in the ER/Golgi or trans-Golgi network forming hexameric assemblies known as hemichannels or connexons, which are delivered to the plasma membrane by vesicular carriers travelling along microtubules [2–4]. Plasma membrane hemichannels form aqueous conduits with a pore diameter of ∼1.5 Å and open in response to various types of stimuli, including mechanical, shear, ionic and ischemic stress [3,4]. Open hemichannels provide a pathway for the release from cells of ATP, glutamate, NAD+ and prostaglandin E2, which act as paracrine messengers [3,4]. Intercellular (gap junction) channels are formed by the head-to-head docking of two connexons in adjacent cells and mediate the exchange of a variety of cytoplasmic molecules (virtually all soluble second messengers, amino acids, nucleotides, ions, glucose and its metabolites) [2]. Connexin mutations that alter either hemichannel or gap junction function have been implicated in a variety of human diseases [5].

It is well established that hemichannel activity is inhibited at normal (∼1 mM) [Ca^2+^]_e_ and this may serve as a protecting mechanism against the harmful effects of leaky hemichannels [3,4,6–8]. Lowering [Ca^2+^]_e_ to micromolar levels fosters hemichannel opening and promotes signaling [9]. Atomic force microscopy imaging shows significant and reversible changes of pore diameter at the extracellular mouth of Cx26 hemichannels exposed to different [Ca^2+^]_e_ [10]. Also the voltage gating of connexin hemichannels is affected by [Ca^2+^]_e_. Lowering [Ca^2+^]_e_ to zero causes a marked increase in the amplitude of hemichannel currents, shifts their activation to more negative potentials and alters the kinetics of activation and deactivation, whereas increasing [Ca^2+^]_e_ has opposite effects (reviewed in Ref. [11]). Molecular dynamics (MD) simulations [12] show that multiple Ca^2+^ ions may linger within the negatively charged extracellular mouth of Cx26 hemichannels at a membrane potential of −80 mV, and low-affinity binding of several Ca^2+^ ions near the point of narrowest pore constriction can occlude the pore. Upon depolarization to 0 mV the interactions weaken and Ca^2+^ ions shift towards the extracellular space [12]. Asp46 and Glu47, two highly conserved negatively charged amino acids facing the pore in the extracellular mouth, are strong candidates for Ca^2+^ binding [13]. Glu47 is also a candidate for gamma carboxylation [14], a post-translational modification that would increase its capability of coordinating Ca^2+^.

**Fig 1:**
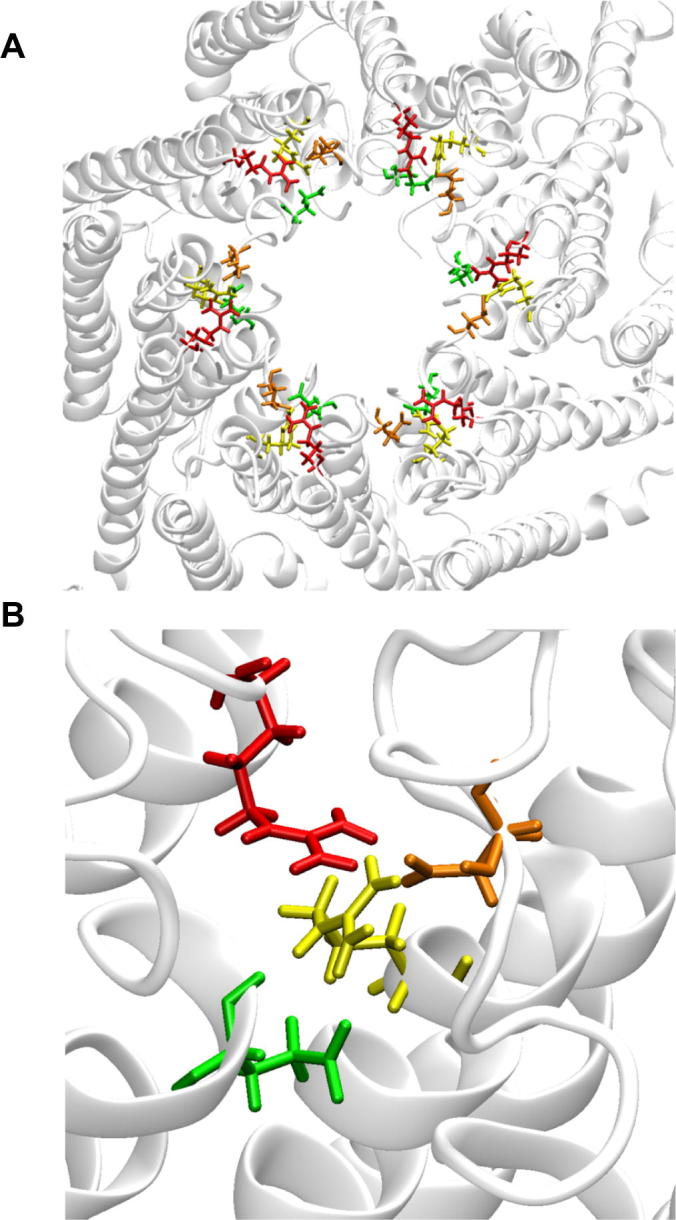
(A) Cx26 hemichannel view from the extracellular region. The two extracellular loops connecting the four transmembrane helix are not shown for clarity. Protein backbone is shown in ribbon representation, while residues mentioned in the text are shown in licorice representation: color legend: γGlu42 (green), γGlu47 (orange), Arg75 (yellow), Arg184 (red). (B) Close up view of the residues interacting with γGlu47. Part of two connexins protomers are shown in ribbons. The configuration is taken from an equilibrium MD trajectory, in which in positions 42 and 47 there were two standard (non gamma carboxylated) Glu. (For interpretation of the references to color in this figure legend, the reader is referred to the web version of this article.)

According to the crystal structure of Cx26 [15], Glu47 is found in the middle of the parahelix formed by residues 42–50 at the border between transmembrane helix 1 (TM1) and the first extracellular loop (E1). MD simulations of the Cx26 hemichannel suggest that Glu47, Arg75 and Arg184 are components of an extensive intra/intersubunit electrostatic network that includes salt-bridge and hydrogen-bond formation and stabilizes the parahelix and TM1/E1 bend angle (Fig. 1) [16].

Arg75 and Arg184 are conserved among different connexin isoforms and the deafness-associated R75Q and R75W mutations of Cx26 produce proteins that prevent the formation of functional gap junction channels [17–19]. The absence of gap-junctional communication caused by R75W expression is due to defective gap-junction formation by functional hemichannels [20]. In Cx32, mutations of Arg75 (R75Q, R75P, R75W) associated with X-linked Charcot Marie Tooth disease (CMT1X) result in trafficking problems, and mutant proteins fail to reach the plasma membrane [21]. A pathogenic role has been also attributed to mutations of Arg184 in Cx26 (R184P [22], R184Q [23], R184W [24]), as well as to mutation of the homologous Arg183 in Cx32 (R183C, R183H, R183S [25]).

Here, we used quantum chemistry computations to investigate the effects exerted by coordination of Ca^2+^ to γGlu47. We examined the equilibrium configuration of a local cluster of amino acids surrounding γGlu47, including Arg75 and Arg184, and singled out a local rearrangement of arginines that can be linked to one of the hemichannel gating mechanisms.

## 2 Materials and methods

All geometries (γGlu47, dyads and triads with and without Ca^2+^) were fully optimized *in vacuo* at the B3LYP/6-31G(d,p) level of theory [26,27] as implemented in the software package Gaussian [28]. The initial structures were derived from an equilibrated MD configuration of the Cx26 connexin hemichannel [12,29] and Glu residues were gamma carboxylated before geometry optimization; a Ca^2+^ was subsequently added and the structure re-optimized. Single point energies and binding energies were computed using a larger basis set, i.e. B3LYP/6-311++G(d,p). Binding energies Δ*E* were computed by partitioning the molecular system into two suitable fragments (fragments are labeled as contiguous sequences of amino acids (AA) without hyphenation to distinguish them from dyads and triads and are not in bold); the sum of the energies of each fragment, calculated separately without relaxing their geometries, has been subtracted to the energy of the total system; according to this definition a negative Δ*E* corresponds to a favorable binding. Basis set superposition error (BSSE) [30], although negligible when neutral fragments are involved, was evaluated and taken into account in the results. The amino terminus of the isolated γGlu was saturated with an acetyl group replacing H, while the carboxylic group was saturated with a NHCH_3_ group replacing OH. In all other larger molecular systems, the amino and carboxyl termini were described simply as –NHCH_3_ and –COOCH_3_, respectively, to reduce the computational effort. Unless otherwise stated, γGlu residues were always considered dianions.

A two-layer QM/QM approach was set up for full geometry optimization of a larger AA cluster formed by 15 residues (γGlu42-Val43-Trp44-Gly45-Asp46-γGlu47-Gln48-Ala49-Phe51-Tyr65-Ser72-Arg75-Val182-Arg184-Lys188), using the ONIOM (Our own N-layered Integrated molecular Orbital + Molecular mechanics) scheme implemented in Gaussian [31]. The inner layer (or high layer) was accurately described using the density functional theory (DFT) method; B3LYP functional and 6-31G(d,p) basis set were employed. This layer includes part of the side chain of γGlu47 $\left({\left(\text{COO}\right)}_{2}{\text{CHCH}}_{2}^{2-}\right)$ and part of the side chain of arginines $\left({\left({\text{NH}}_{2}\right)}_{2}\text{C}(\text{NH}){\text{CH}}_{2}^{+}\right)$, and Ca^2+^ ion when present. The PM3 hamiltonian [32] was used for the outer layer (or low layer), which is formed by the residues located within 4 Å from γGlu47 of the inner layer (γGlu42-Val43-Trp44-Gly45-Asp46-Gln48-Ala49-Phe51-Tyr65-Ser72-Val182-Lys188). This computational approach has been satisfactorily used before by some of us for structural investigation of different protein systems [33,34]. Single point and binding energies were calculated also on the triads γGlu47-Arg75-Arg184 (with and without Ca^2+^), extracted from ONIOM optimized structures at the B3LYP/6-311++G(d,p) level of theory.

## 3 Results and discussion

Our working hypothesis is that the peculiar and specific interaction of Ca^2+^ with a gamma carboxylated glutamate, in particular γGlu47, may impart distortions in the structure of Cx26 by modifying the interaction between γGlu47 and two proximal arginines (Arg75 and Arg184) in the AA cluster γGlu47-Arg75-Arg184. To investigate the binding mode of Ca^2+^, a bottom-up approach was undertaken, based on state-of-the-art quantum chemistry methods, i.e. hybrid DFT calculations. The first step was the full (unconstrained) geometry optimization of a non-coded γGlu. A monoanionic structure, characterized by a neutral (protonated) and an anion carboxylate adjacent group, was fully optimized at the B3LYP/6-31G(d,p) level of theory. The calculation converged to the same molecular geometry (Supplementary Fig. 1A), irrespective of the choice of the carboxylate group which can be initially protonated. In the T-shaped structure the carboxylate groups are arranged in a five-membered ring fashion; the angle O–H–O is close to 160° and the O–H distances are 1.04 and 1.46 Å, respectively.

The optimization of the dianionic form, with both negatively charged adjacent carboxylates, converged, but involved a proton transfer from the N terminus to one carboxylate group (Supplementary Fig. 1B). In this case the T-shaped form is preserved but the five membered ring is disrupted. Thus the dianion form with both negative charges on the carboxylate groups is unlikely a standalone species, but in the protein environment where protonated arginines are present in close proximity, it is reasonable to treat γGlu residues as dianions, which are also better candidates for binding calcium. In the optimized γGlu-Ca^2+^ geometry (Supplementary Fig. 1C), the T-shaped structure is retained and the ion is chelated by three O atoms of the two negatively charged carboxylates; the average Ca–O distance is 2.3 Å; the fourth O atom is hydrogen bonded to the close amide group and cannot be involved in calcium binding for steric reasons. The Δ*E in vacuo* after Ca^2+^ coordination is −512.9 kcal mol^−1^, thus the binding is largely favored (Table 1). It is worth mentioning that this large value is mainly due to electrostatic contributions and is of the same order of magnitude of binding energies reported for analogous systems [35].

**Fig. 2:**
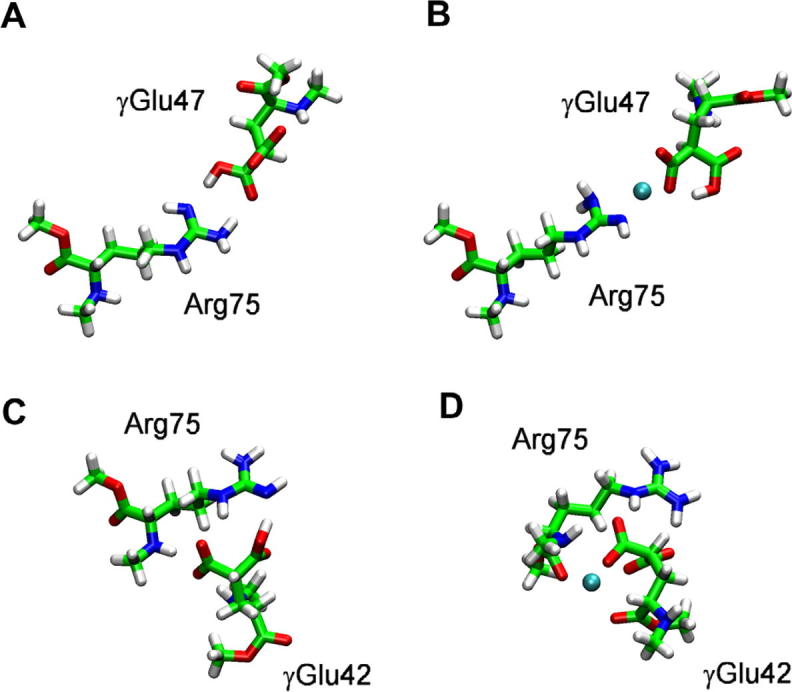
B3LYP/6-31G(d,p) optimized dyads. **γGlu47-Arg75** without (A) and with (B) calcium ion, **γGlu42-Arg75** without (C) and with (D) calcium ion.

Subsequently, two dyads of amino acids were considered, i.e. **γGlu47-Arg75** and **γGlu42-Arg75** (Fig. 2). γGlu42 was examined because Glu42 is close to Glu47, it is another candidate for gamma carboxylation and it too, in principle, could bind Ca^2+^, although it is not conserved in all connexin isoforms. Both Glu residues face the amino groups of Arg75 and in both dyads, a proton transfer occurs during geometry optimization from Arg75 to one of the carboxylate groups of glutamate, which becomes a monoanion (Fig. 2).

In the presence of a calcium ion, the two γGlu residues behave very differently (Fig. 2B, D). γGlu42 uses its acetyl tail together with its carboxylate groups to bind Ca^2+^ (Fig. 2D), however this imposes a severe distortion to its structure, which is highly unlikely to occur in the protein. By contrast, in the dyad **γGlu47-Arg75** with Ca^2+^ (Fig. 2B), the ion is chelated by one COO^−^ group of γGlu47 and two N atoms of Arg75, both having their electron pair available after a proton transfer from one of them to the second carboxylate group of glutamate. The binding energy between γGlu47 and Arg75 is −14.7 kcal mol^−1^, a value which accounts mainly for salt bridge formation since the total charges of the fragments are −1 and 0, respectively. As expected, the binding energy of Ca^2+^ to the γGlu47Arg75 fragment is much larger, i.e. −379.9 kcal mol^−1^, due to the dominant electrostatic contribution (Table 1).

**Fig. 3:**
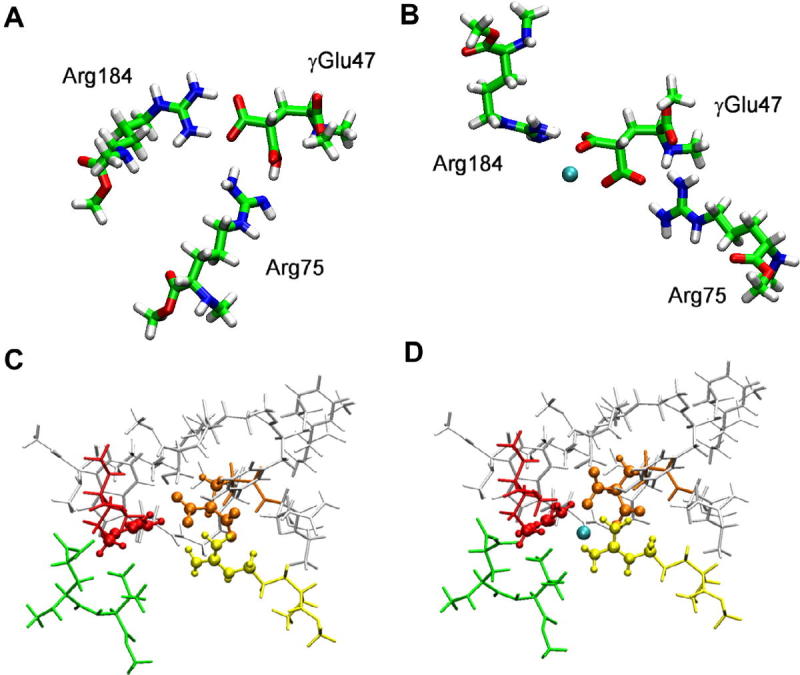
B3LYP/6-31G(d,p) optimized triads **γGlu47-Arg75-Arg184** without (A) and with (B) calcium ion. Cluster of AA optimized at B3LYP/6-31G(d,p):PM3 level, without (C) and with (D) calcium ion; ball and stick representation is used for the high layer, licorice representation is used for the low layer. Color code for the residues in panels C and D, are the same of Fig. 1: γGlu42 (green), γGlu47 (orange), Arg75 (yellow), Arg184 (red). (For interpretation of the references to color in this figure legend, the reader is referred to the web version of this article.)

We finally examined the AA triad **γGlu47-Arg75-Arg184** (Fig. 3A, B; recall that Arg184, which can interact with γGlu47, belongs to an adjacent connexin). The salt bridge between γGlu47 and Arg75 is maintained in the isolated triad; the second COO^−^ is protonated after a proton transfer from Arg184 (Fig. 3A). Note that the backbone of Arg184 is significantly displaced from its initial position, however this rearrangement is unlikely to occur in the protein due to the interactions with the surrounding amino acids. In the presence of Ca^2+^ (Fig. 3B) the salt bridge is preserved, although slightly distorted. As in the dyad **γGlu47-Arg75,** Ca^2+^ interacts with three O atoms of γGlu47 and two N atoms of Arg75 (Fig. 3B). On the basis of the binding energies computed using two different fragmentations, i.e. γGlu47Arg75 and Arg184 and γGlu47Arg184 and Arg75, we conclude that the interaction between γGlu47 with Arg75 (−111.8 kcal mol^−1^) is stronger than the interaction with Arg184 (−18.3 kcal mol^−1^). However, the in presence of Ca^2+^, the ion is chelated by γGlu47, the binding of the latter to Arg184 becomes less favorable, and the binding of γGlu47 to Arg75 is neatly unfavorable (−4.6 and +17.3 kcal mol^−1^, respectively). This strongly suggests that Ca^2+^ can alter the local structure, because its binding to γGlu47 prevents any stabilizing interaction between γGlu47 and the close arginines.

To further validate these results, obtained for the model triad *in vacuo*, and to take into account properly the protein environment, we constructed a two-layer ONIOM model in which the core formed by portions of γGlu47-Arg75-Arg184 (with and without Ca^2+^) is accurately described at B3LYP/6-31G(d,p), and the surrounding amino acids within a sphere centered on γGlu47 with a radius of 4 Å are treated using the PM3 hamiltonian (Fig. 3C and D). The geometry of the two-layer ONIOM model was fully optimized (without any constraint). Note that only portions of the three amino acids (and Ca^2+^, when present) were included in the inner layer, as explained in Section 2, and shown in ball and stick representation in Fig. 3C and D. In the triad γGlu47-Arg75-Arg184 extracted from the ONIOM model, the salt bridge between γGlu47 and Arg75 is maintained and no proton transfer from Arg184 to γGlu47 takes place. Indeed, analysis of the binding energies shows the Δ*E* is much larger, due a stronger electrostatic contribution for this system than for the model triad discussed above (−130.4 vs −18.3 kcal mol^−1^; see Table 1). In the presence of Ca^2+^ (Fig. 3D), the salt bridge is destroyed and Ca^2+^ binds two oxygen atoms, belonging to each COO^−^ group of γGlu47, as in the isolated triads (Fig. 3B). Importantly, this binding mode is identical to that observed in the isolated γGlu47-Ca^2+^ complex. As in the isolated triad, energy decomposition analysis reveals that the interaction between γGlu47 and the close arginines is disrupted (Table 1). This result supports the idea that a local bond rearrangement, i.e. binding of Ca^2+^ to γGlu47 and the breaking of salt bridges between γGlu47 and the neighboring arginines impacts on the energetics and induces a structure rearrangement in a critical region of connexin hemichannels.

To conclude, our results suggest that the post-translationally gamma carboxylated Glu47 is a strong candidate to coordinate Ca^2+^ in the extracellular vestibule of a Cx26 hemichannel. A consequence of this putative coordination action is a rearrangement of the side chains of two highly conserved arginines (Arg75 and Arg184), that are required for the correct functioning of connexin hemichannels and gap junction channels, accompanied by large energetics variations. The salt bridges between Glu47 and Arg184 are considered important to maintain the quaternary structure of the connexin hemichannel [36,37], and Glu47 is part of the parahelix that is thought to undergo a structural rearrangement to close the hemichannel in response to membrane hyperpolarization and extracellular calcium [38]. More extensive computations and simulations are currently underway to assess whether this distortion is sufficient to initiate the experimentally observed gating processes.

## Figures and Tables

**Table 1:** Binding energies Δ*E* between defined fragments of the studied model compounds; total charges are given in parenthesis. All energies include BSSE correction.

Compound	Fragments	Binding energy Δ*E* (kcal mol^−1^)
**γGlu-Ca^2+^** (0)	γGlu (−2) Ca^2+^ (+2)	−512.9
**γGlu47-Arg75** (−1)	γGlu47 (−1) Arg75 (0)	−14.7
**γGlu47-Arg75-Ca^2+^** (+1)	γGlu47Arg75 (−1) Ca^2+^ (+2)	−379.9

**γGlu47-Arg75-Arg184** (0)	γGlu47Arg75 (0) Arg184 (0)	−18.3
	γGlu47Arg184 (−1) Arg75 (+1)	−111.8

**γGlu47-Arg75-Arg184-Ca^2+^** (+2)	γGlu47Arg75Arg184 (0) Ca^2+^ (+2)	−271.3
	γGlu47Arg184Ca^2+^ (+1) Arg75 (+1)	17.3
	γGlu47Arg75Ca^2+^ (+1) Arg184 (+1)	−4.6

**γGlu47-Arg75-Arg184** (0)^a^	γGlu47Arg75 (−1) Arg184 (+1)	−130.4
	γGlu47Arg184 (−1) Arg75 (+1)	−88.6

**γGlu47-Arg75-Arg184-Ca^2+^** (+2)^a^	γGlu47Arg75Arg184 (0) Ca^2+^ (+2)	−229.3
	γGlu47Arg184Ca^2+^ (+1) Arg75 (+1)	+33.2
	γGlu47Arg75Ca^2+^ (+1) Arg184 (+1)	+20.6

a Optimized geometries extracted from ONIOM model.

## Appendix A Supplementary data

Supplementary data 1 This document contains supplementary data.
