# Supplementary material for: Role of gamma carboxylated Glu47 in connexin 26 hemichannel regulation by extracellular Ca2+: Insight from a local quantum chemistry study
Source: Biochem Biophys Res Commun. 2014 Feb 28;445(1):10–5. doi: 10.1016/j.bbrc.2014.01.063 (PMC3969289; doi:10.1016/j.bbrc.2014.01.063)
Supplement: Supplementary data 1 — This document contains supplementary data. [file mmc1.docx]

**Supporting Information**

**Role of gamma carboxylated Glu47 in connexin 26 hemichannel regulation by extracellular Ca^2+^: insight from a local quantum chemistry study**

**Francesco Zonta,^a^ Fabio Mammano,^a,b,c*^**

**^a^** *Dipartimento di Fisica e Astronomia “G. Galilei”, Università di Padova, 35131 Padova, Italy*

*^b^ Istituto Veneto di Medicina Molecolare, Fondazione per la Ricerca Biomedica Avanzata, 35129*

*Padova, Italy*

*^c^ Istituto CNR di Neuroscienze, 35131 Padova, Italy*

**Mauro Torsello,^d^ Nicola Fortunati,^d^ Laura Orian^d*^ and Antonino Polimeno^d^**

***^d^*** *Dipartimento di Scienze Chimiche, Università degli Studi di Padova Via Marzolo 1 35129 Padova Italy*

[*****Dr. Laura Orian:](mailto:*Dr.%20Laura%20Orian:%20laura.orian@unipd.it) [laura.orian@unipd.it](mailto:laura.orian@unipd.it); *****Prof. Fabio Mammano: fabio.mammano@unipd.it

**Figure S1** B3LYP/6-31G(d,p) optimized **γ**Glu (charge: -1 (**A**) and -2 (**B**)), **γ**Glu with calcium ion (**C**).

| 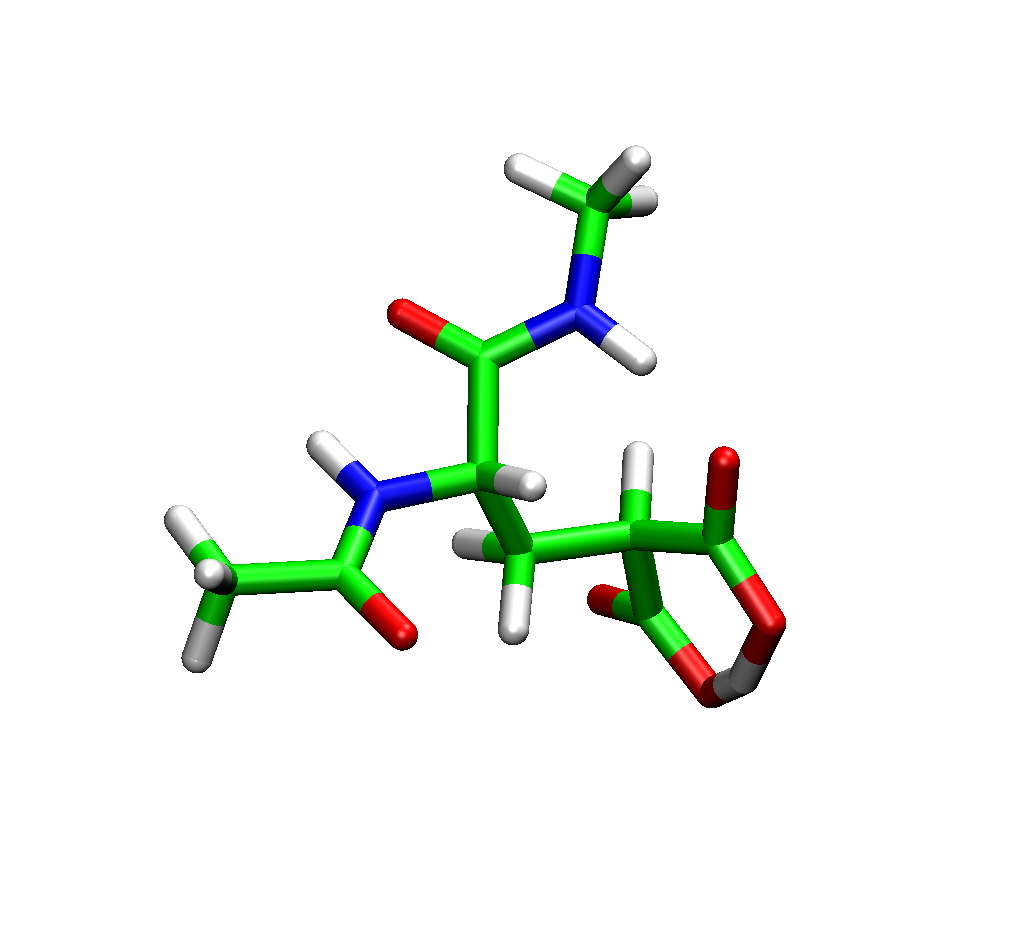 | 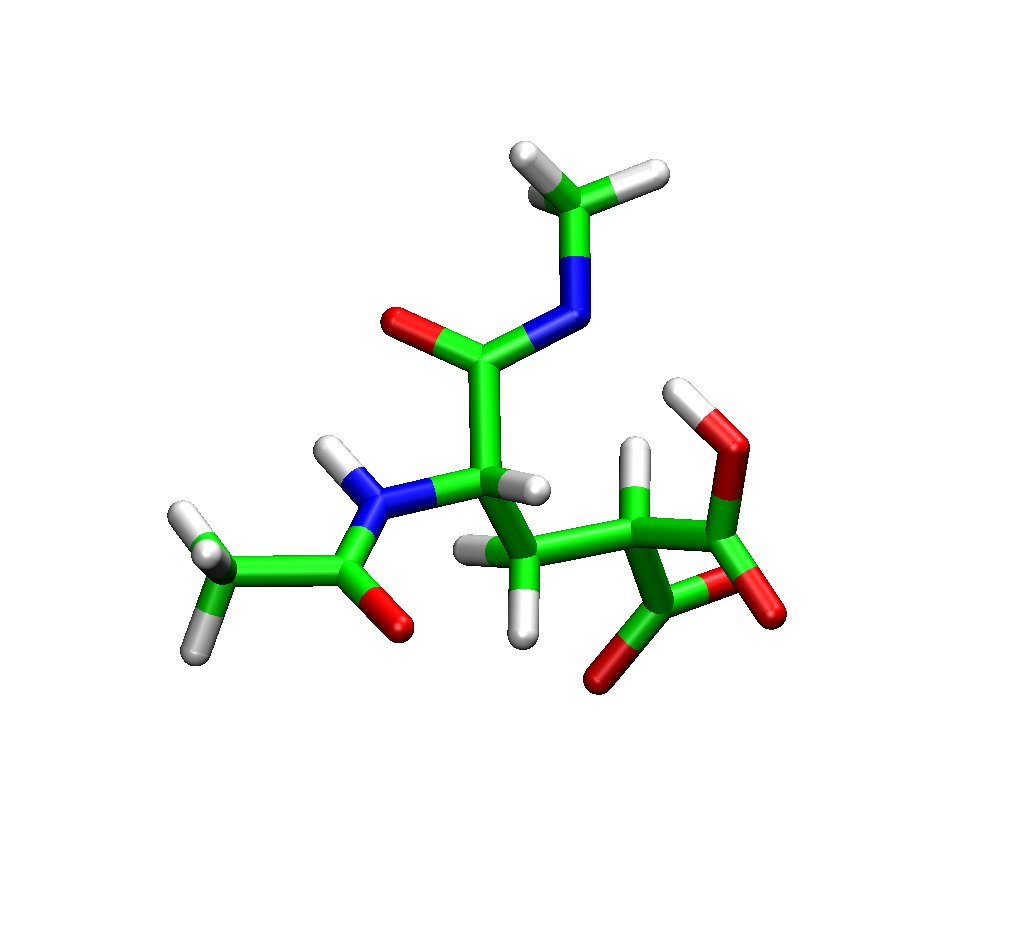 | 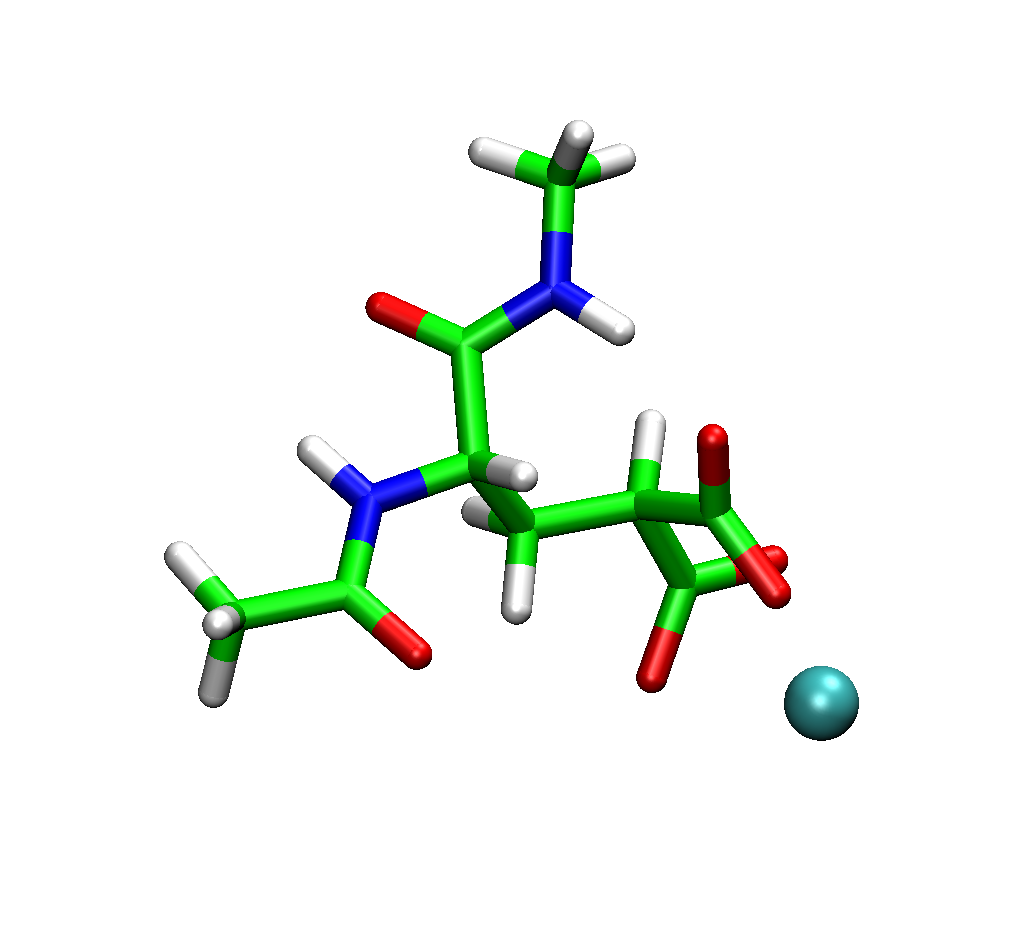 |
| --- | --- | --- |
| **A** | **B** | **C** |

**Figure S2** Triad γGlu47-Arg75-Arg184 without (**A**) and with (**B**) calcium ion extracted from ONIOM model.

| **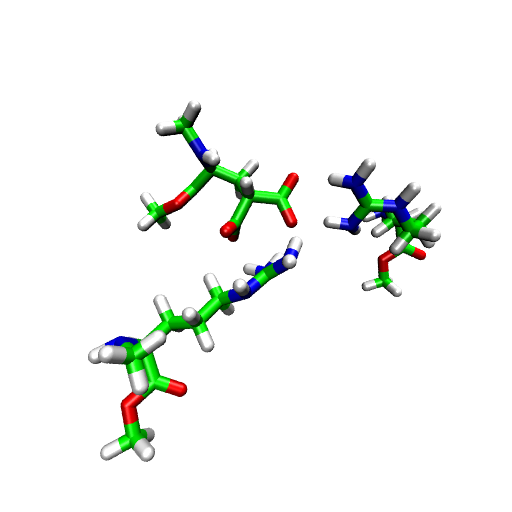** | **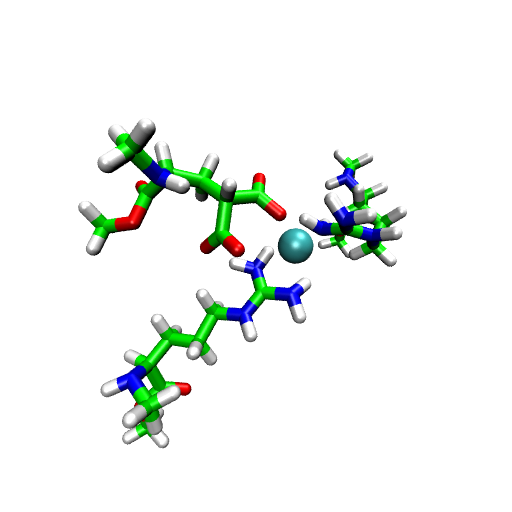** |
| --- | --- |
| **A** | **B** |

**γGlu42-Val43-Trp44-Gly45-Asp46-γGlu47-Gln48-Ala49-Phe51-Tyr65-Ser72-Arg75-Val182-Arg184-Lys188**

AA cluster used in ONIOM calculation (without Ca^2+^ ion).

N 53.51286200 44.91653700 69.56330200

H 54.34513300 44.41873200 69.79838600

C 53.06714200 45.74878200 70.71471200

H 52.22967200 46.40686700 70.36036400

C 52.52036900 44.85835300 71.84326900

H 53.04596400 43.88135900 71.87041800

H 52.70954300 45.34416900 72.83150100

C 51.01341400 44.63146600 71.74557300

H 50.75274600 44.26836000 70.72361400

C 50.21642700 45.93902500 72.00622300

O 49.02427100 46.03393200 71.63309700

O 50.76300100 46.92625000 72.59008000

C 54.19134100 46.67639800 71.21695800

O 54.35596200 47.81581400 70.81469100

N 55.17298400 46.15849700 72.13723400

H 54.92527900 45.25715500 72.50683000

C 55.66171000 47.03573300 73.23145700

H 55.42009900 48.10234900 72.97686100

C 55.03007700 46.72434400 74.61367000

H 55.60595500 47.30879500 75.37295100

C 53.58322900 47.19187900 74.65003400

H 53.49756100 48.26991200 74.45998400

H 52.94692800 46.67365400 73.90466100

H 53.12542600 46.99535300 75.63578900

C 55.10402300 45.25145200 74.98487300

H 54.57733000 45.06056400 75.92916700

H 54.63397900 44.61542200 74.21510700

H 56.14031500 44.91188500 75.10772400

C 57.18857600 46.95221100 73.33087500

O 57.94361000 47.87181400 73.58692700

N 59.62144300 43.02620000 85.34034500

H 60.32690300 43.13475800 86.03794700

C 58.93463800 41.72335200 85.55587100

H 58.78091700 41.66247900 86.66678500

C 57.52767000 41.67824200 84.95036800

H 57.03405800 40.75096200 85.31173000

H 56.94034100 42.51565000 85.38906700

C 57.41879800 41.74220800 83.43402600

H 57.79713800 42.72527900 83.07092300

H 58.06634600 40.97880100 82.95870700

C 55.96601700 41.53145800 83.02914400

H 55.67508800 40.47868200 83.18577700

H 55.27304000 42.18048100 83.60158800

N 55.77827000 41.83729500 81.59079200

H 56.20843700 42.71243500 81.31705100

C 54.54787300 41.67529600 81.04486500

N 53.63281300 40.87087000 81.61898700

H 53.48274800 41.05309800 82.62085900

H 52.72046800 41.00101700 81.17134500

N 54.27957900 42.18934200 79.83654900

H 54.95379700 42.80860500 79.41582000

H 53.28206400 42.39217700 79.69069100

C 59.79496200 40.50977700 85.18597100

O 59.58822700 39.61064800 84.39924100

N 51.07109100 49.12687300 76.92947900

H 51.90520600 49.11380000 76.38042800

C 51.10302500 48.07298400 77.97467800

H 50.23350100 48.24931900 78.66943600

C 50.94109600 46.69411200 77.30647800

H 51.82503000 46.49677500 76.65371700

H 50.95658100 45.92464000 78.10719500

C 49.71881300 46.51194200 76.50115300

C 49.64571100 46.64107300 75.11699100

H 50.41403000 46.90902700 74.33027600

N 48.37048500 46.27715500 74.68923200

H 48.13724900 46.08923700 73.73989400

C 47.61616300 45.86812400 75.80495700

C 46.30352300 45.38566100 75.89928600

H 45.68555900 45.26375200 75.00412300

C 45.83371700 45.05783300 77.15398400

H 44.81863500 44.66685400 77.27054100

C 46.64790900 45.18893900 78.29540500

H 46.24974500 44.88063700 79.27288400

C 47.94324300 45.65985800 78.21399800

H 48.57998800 45.72014700 79.10646500

C 48.44817700 46.02155400 76.95778400

C 52.40873600 48.05704700 78.80654800

O 53.10773300 47.06928400 78.96576900

N 52.88982600 49.28729100 79.36035500

H 52.17501300 49.97546500 79.51221800

C 53.95648300 49.29011900 80.38044100

H 54.57898300 48.37373900 80.29348600

H 54.62100200 50.14040300 80.11993900

C 53.51821000 49.42460300 81.83570500

O 54.21065700 50.02279600 82.65187900

N 52.34066500 48.74529800 82.25824500

H 51.60554300 48.74481200 81.55092700

C 51.74189600 49.08505600 83.57885700

H 52.57345300 49.26476300 84.31913400

C 50.88072700 50.35824200 83.50499200

H 50.29631200 50.47143600 84.44040700

H 51.54031800 51.24800800 83.45307600

C 49.94424500 50.41558000 82.31497900

O 50.17312600 49.80728900 81.21689700

O 48.86352700 51.07014700 82.28866000

C 50.94649300 47.89856400 84.15279400

O 50.12850500 48.05206200 85.05750000

N 51.23065300 46.59136400 83.73427300

H 51.76607500 46.42698500 82.90145300

C 50.42736400 45.42606400 84.17712900

H 49.39716400 45.79676400 84.46662400

C 50.21143200 44.38522100 83.06295200

H 49.90999400 43.42059100 83.51653300

H 49.37484100 44.71955700 82.40797100

C 51.40990500 44.08692900 82.12842900

H 51.83543300 45.03011500 81.74260100

C 50.89355100 43.34569900 80.84688100

O 49.83748800 43.79008100 80.32591600

O 51.57130900 42.37061100 80.38439000

C 51.01989100 44.77347300 85.44321700

O 50.30250000 44.28397900 86.30458500

N 52.43157600 44.82771000 85.65500600

H 52.94795000 44.84000100 84.79665300

C 53.06617700 43.95267200 86.68056700

H 52.27941000 43.68043600 87.43447900

C 53.66444900 42.67019500 86.08214300

H 53.12709400 42.41496000 85.10467900

H 54.72399800 42.82174700 85.78661700

C 53.55347600 41.46211300 86.99618800

H 52.49659800 41.33352800 87.33446000

H 53.77447300 40.55218800 86.40029400

C 54.51333500 41.50660100 88.16729300

O 55.69425700 41.18105100 88.09966400

N 54.00521900 41.84623100 89.44619100

H 54.69172700 42.12274000 90.11289900

H 53.23128900 42.47352600 89.41251300

C 54.14030600 44.77823600 87.41106100

O 54.14761400 44.90837500 88.62896200

N 55.22055900 45.30763200 86.65759400

H 55.03444700 45.48512100 85.69301100

C 56.20095200 46.24193700 87.26167500

H 56.42440600 45.87401300 88.29575100

C 57.48245700 46.23435100 86.43409900

H 57.89170400 45.21810200 86.33965700

H 57.32617400 46.62080900 85.41718400

H 58.25241200 46.85715500 86.90766000

C 55.69660700 47.68559500 87.37993800

O 55.92533800 48.45688900 88.29098200

N 51.37413800 47.58559500 88.98672900

H 52.24610500 47.39632100 89.43498000

C 50.24181000 47.10777400 89.82286900

H 49.29430700 47.41510800 89.30853800

C 50.29196400 45.57108000 89.87801300

H 50.29508500 45.19397200 88.83109000

H 51.24089800 45.22622600 90.33890600

C 49.12178600 44.99529000 90.60828800

C 47.88461400 44.87684600 89.97218200

H 47.76554200 45.21015200 88.92652200

C 46.80140300 44.32792100 90.64856400

H 45.83567700 44.23588200 90.14051400

C 46.94339500 43.89407400 91.96245900

H 46.08863700 43.46367300 92.49313400

C 48.17568000 44.00279800 92.59737100

H 48.29297400 43.65466100 93.62826300

C 49.26261400 44.54831100 91.92359500

H 50.23422800 44.62761000 92.42549500

C 50.28140100 47.70308000 91.23475500

O 51.18771000 47.67127600 92.04384200

N 48.78879000 39.78233600 90.16608400

H 48.82931500 38.90374000 90.63836300

C 48.64739500 39.57003300 88.69955500

H 48.58285700 40.58299100 88.22344900

C 49.90119200 38.85487900 88.17117900

H 50.80310200 39.33105900 88.61066300

H 49.92374200 37.79896300 88.50935900

C 49.99987700 38.92082500 86.68184800

C 50.57555100 40.03936200 86.07046800

H 50.93475100 40.87729500 86.69100400

C 50.72756100 40.10761000 84.69375300

H 51.19625700 41.01085700 84.22022200

C 50.29415800 39.01924000 83.92021300

O 50.52892400 39.09819900 82.57517400

H 49.99946100 38.43663400 82.14557900

C 49.69612400 37.89992200 84.51423400

H 49.33538400 37.06423500 83.90405400

C 49.55507200 37.85966700 85.89567800

H 49.09263100 36.98517100 86.36714600

C 47.36976900 38.80093200 88.33882700

O 46.42686800 39.20233100 87.68971800

N 46.87314300 40.06128400 83.21277600

H 47.83333200 40.02446100 83.49240500

C 46.74602500 40.70579300 81.86743600

H 46.18927200 41.68655600 81.94252700

C 48.12424700 40.99120500 81.22393400

H 48.79688700 41.47938300 81.95978900

H 48.60991500 40.05316200 80.89077200

O 47.96289700 41.80664000 80.09701800

H 48.60426500 42.52389100 80.19181700

C 45.94407200 39.71606500 81.02399000

O 46.30170000 38.66129600 80.53708900

N 46.12758200 38.58049400 77.00836000

H 46.54627400 38.53815200 77.91518000

C 47.14591000 38.38657300 75.94492500

H 46.62713700 38.56424000 74.96826000

C 48.25466000 39.42977200 76.12663700

H 47.79006000 40.40646200 76.38047700

H 48.89160400 39.16823600 77.00273000

C 49.10696000 39.58840800 74.87680500

H 49.34607700 38.59261100 74.45124800

H 48.52784300 40.11579300 74.09034900

C 50.42244300 40.31428900 75.12935500

H 51.06122100 39.77273400 75.84359100

H 50.98082000 40.39367400 74.17786900

N 50.25131100 41.69244600 75.61191100

H 50.11833000 42.44973000 74.86839600

C 50.38942700 42.05008900 76.87343300

N 50.59217100 41.11713600 77.86000500

H 49.95605000 40.32237500 77.84325800

H 50.72820400 41.52350400 78.79818400

N 50.43584500 43.34667700 77.21875300

H 50.30669200 44.08025900 76.52590900

H 50.18343700 43.57931400 78.18674100

C 47.72824700 36.96610300 75.90591300

O 47.66185600 36.17228400 74.98758400

N 46.26191900 45.64364500 85.52368400

H 45.27450500 45.70118300 85.65841200

C 46.64936500 46.28295800 84.23800200

H 47.77681100 46.34681200 84.23475700

C 46.20388900 45.43563500 83.02235400

H 45.10647900 45.57738700 82.86630000

C 46.95208400 45.88860700 81.77445300

H 46.57214200 45.37401600 80.87438200

H 46.86304300 46.97210800 81.58850300

H 48.03106200 45.65217400 81.84089100

C 46.47747200 43.95510300 83.22565800

H 47.54272100 43.76795800 83.43480100

H 45.90532000 43.50861400 84.05596500

H 46.22027900 43.37727400 82.31919900

C 46.03361400 47.68380600 84.11840100

O 44.89584100 48.01721500 84.40059300

N 46.96022900 50.30881800 74.37329000

H 46.51934100 51.19641600 74.27213700

C 45.97169700 49.26465900 74.73615800

H 46.45588700 48.26834300 74.54939800

C 45.60637200 49.35362900 76.22508000

H 45.10520400 50.31375200 76.45457200

H 44.86100600 48.56298200 76.44481200

C 46.82354700 49.17176700 77.11921100

H 47.51964500 48.41611800 76.68446800

H 47.40560100 50.11530400 77.15769200

C 46.41755900 48.70549200 78.51023200

H 45.52393000 49.25373700 78.87360800

H 46.11033200 47.63451600 78.44945200

C 47.56310600 48.79852600 79.50865100

H 47.37038700 48.12198400 80.38356500

H 48.51995000 48.41841700 79.05182600

N 47.77023900 50.20241400 80.00190300

H 48.56412900 50.26550600 80.70624800

H 47.97931400 50.79623800 79.22108500

H 46.92788400 50.52816700 80.43836000

C 44.66603900 49.29500800 73.93119000

O 43.99889900 48.34846300 73.57593800

O 54.94963500 48.10599400 86.32030500

C 54.39425100 49.40239600 86.34416900

H 53.63620400 49.49438600 87.13784700

H 55.15555600 50.18029400 86.46001600

H 53.91927100 49.47009600 85.35578300

O 49.13248800 48.36380900 91.57133900

C 49.02621200 48.93752100 92.85449800

H 49.10494000 48.18472100 93.64623700

H 49.77635500 49.71948900 93.01480600

H 48.02289000 49.37043400 92.83978500

O 46.89722000 48.62050600 83.64487900

C 46.48988600 49.96732300 83.57067100

H 45.66447400 50.09958000 82.86256800

H 46.19569600 50.36076700 84.54851300

H 47.41505900 50.46251100 83.20277400

O 44.64156000 40.10314200 80.85520200

C 43.75713100 39.24827800 80.16768900

H 44.10130000 38.99737800 79.15154000

H 43.58246800 38.32282100 80.72741400

H 42.84019400 39.84091600 80.11963700

O 47.30869700 37.53135600 88.85288200

C 46.19496000 36.72620700 88.53506900

H 45.26236700 37.15129100 88.92170000

H 46.10640800 36.56094100 87.45580800

H 46.42699800 35.78885100 89.04677300

O 60.96091300 40.46588400 85.91120000

C 61.85094800 39.39186100 85.70695900

H 61.39074200 38.42810200 85.95043700

H 62.23263900 39.36966800 84.68054700

H 62.65807100 39.61674800 86.40876300

O 48.36913300 36.58301800 77.04985100

C 48.95015400 35.29981800 77.10913100

H 48.20026700 34.50798600 77.00685700

H 49.72972100 35.16879700 76.35094100

H 49.38562400 35.28482300 78.11146500

O 44.20488300 50.54835300 73.62628800

C 43.03009600 50.66838800 72.85691700

H 43.13508900 50.20351400 71.87249300

H 42.16196400 50.24685900 73.37051600

H 42.92258500 51.74902500 72.75690100

O 57.73430800 45.71620400 73.12759100

C 59.13540300 45.57573300 73.18941400

H 59.64396600 46.18615000 72.43546100

H 59.52733700 45.81860600 74.18310400

H 59.27618000 44.51324800 72.97525600

C 47.77522400 49.98546600 73.18936000

H 47.19335700 49.88897100 72.26075000

H 48.51347800 50.78426300 73.05220900

H 48.32296400 49.04491900 73.34965400

C 46.98585600 46.17840200 86.69022700

H 46.74140400 47.22401500 86.93205300

H 46.74982900 45.55802500 87.57104100

H 48.07240300 46.11210300 86.52114500

C 53.69394500 45.65602800 68.30421000

H 54.46123500 46.44468100 68.34541500

H 53.97438100 44.94599700 67.51559400

H 52.74318100 46.12541800 68.01899500

C 60.20967100 43.26376400 84.01161200

H 60.74125100 44.22360900 84.02613800

H 60.91325400 42.48244800 83.68402300

H 59.40304100 43.33064000 83.26319500

C 44.95620700 37.69673600 76.93071700

H 45.18759000 36.62481000 77.02633300

H 44.27229500 37.98003900 77.74863600

H 44.43856300 37.84981700 75.97483500

C 47.75298000 40.64261000 90.76107500

H 46.73056600 40.24357000 90.66995200

H 47.97727700 40.77418400 91.82775800

H 47.77181400 41.63731800 90.28988300

C 46.05726200 40.69735700 84.26205000

H 44.99511200 40.55122900 84.02560800

H 46.26636200 40.20515400 85.22098400

H 46.22637800 41.78566100 84.38397800

C 50.71046100 50.47985500 77.37335200

H 49.63585800 50.49343300 77.61146400

H 51.25029400 50.84663900 78.26486400

H 50.88158100 51.18850700 76.55346700

C 51.28619700 48.99093900 88.56270600

H 50.32068900 49.17742800 88.07265000

H 51.40656300 49.71913300 89.37910800

H 52.07966500 49.17099000 87.81665800

C 52.56488500 43.32863000 82.81605800

O 52.25720300 42.31805100 83.53863200

O 53.73006000 43.74961500 82.61238000

C 50.65899300 43.51880400 72.75174800

O 50.30880100 43.79694800 73.94825500

O 50.75041000 42.30727900 72.44994400

**γGlu42-Val43-Trp44-Gly45-Asp46-γGlu47-Gln48-Ala49-Phe51-Tyr65-Ser72-Arg75-Val182-Arg184-Lys188-Ca^2+^**

AA cluster used in ONIOM calculation (with Ca^2+^ ion).

N 53.66172400 45.12708200 69.71391000

H 54.56846500 44.72353800 69.82114300

C 53.23018700 45.76459700 70.98918900

H 52.36507700 46.44329800 70.76532700

C 52.76616500 44.68370000 71.97388500

H 53.24040700 43.70801000 71.73596600

H 53.11317100 44.95150500 73.00454300

C 51.25841200 44.47310500 72.07891700

H 50.84718900 44.09312300 71.11501500

C 50.46238100 45.73789100 72.49516300

O 49.20658900 45.73980000 72.38001600

O 51.04564000 46.73557300 73.00204200

C 54.34265600 46.63321100 71.61651200

O 54.56785000 47.78302700 71.28573000

N 55.22923700 46.04200300 72.59366100

H 54.91974300 45.13483900 72.89622300

C 55.67629900 46.83730900 73.76253900

H 55.40392000 47.91333300 73.58990700

C 55.04571500 46.40551700 75.11409600

H 55.52598000 47.03973100 75.90067800

C 53.55003500 46.67432800 75.12215100

H 53.31955300 47.72877400 74.91637300

H 53.00505100 46.07029300 74.36657500

H 53.11280300 46.42588600 76.10708900

C 55.27991600 44.94394300 75.47017000

H 54.81856700 44.69531700 76.44179400

H 54.83758100 44.27199200 74.71737500

H 56.34862500 44.70675600 75.54637800

C 57.20631100 46.79341600 73.87058700

O 57.91296900 47.67287600 74.32363000

N 58.93024700 43.12076900 84.94762400

H 59.47899300 43.32263200 85.75704700

C 58.34136200 41.76205600 85.09317300

H 58.10908600 41.65374800 86.18889300

C 56.98416100 41.63973300 84.39152000

H 56.55508100 40.65085400 84.65785200

H 56.30064600 42.39364400 84.85240300

C 56.95352800 41.80705400 82.87646200

H 57.42163500 42.77713300 82.59137300

H 57.56897700 41.01987800 82.39400000

C 55.50823600 41.73270900 82.41787900

H 55.06815700 40.75314700 82.67637100

H 54.88174800 42.52806300 82.86180400

N 55.41303200 41.87571700 80.92855200

H 56.26298500 42.08917000 80.42177400

C 54.26451600 41.82205500 80.27725300

N 53.11581900 41.54195200 80.87201300

H 53.03250300 41.84068000 81.85599800

H 52.26581000 41.77448100 80.35934100

N 54.23260000 42.05320200 78.88781600

H 55.13176000 41.90377000 78.43552100

H 53.53582200 41.45353700 78.44497800

C 59.30454200 40.62375900 84.73884300

O 59.24120900 39.82230200 83.82809700

N 50.97255200 49.04867500 76.93778700

H 51.70305000 48.89243000 76.27499700

C 51.08953900 48.14212300 78.10282400

H 50.28764900 48.44327500 78.83720000

C 50.83043900 46.68563900 77.67623400

H 51.71165300 46.34145600 77.07614500

H 50.66053800 46.11122900 78.61570300

C 49.67830800 46.43815500 76.79912100

C 49.68559800 46.59148400 75.41304600

H 50.49694200 46.90753300 74.69325100

N 48.46266300 46.19046500 74.89621400

H 48.33865400 46.05066000 73.89033400

C 47.65250100 45.74195100 75.95927700

C 46.34489500 45.23385400 75.97189500

H 45.77593700 45.12507700 75.04193300

C 45.80854000 44.88293200 77.19365900

H 44.79077200 44.48323400 77.24616000

C 46.54727500 45.01764900 78.38595100

H 46.09265800 44.71881700 79.34270600

C 47.83435300 45.51324300 78.38535400

H 48.40733600 45.60215100 79.31658700

C 48.40366400 45.89758300 77.16494800

C 52.47582500 48.21321900 78.79281500

O 53.34515800 47.33318000 78.72423000

N 52.82744600 49.38463100 79.46291300

H 52.10977100 50.07904500 79.56173000

C 53.97551300 49.49176200 80.38336000

H 54.69139000 48.66083900 80.21069000

H 54.51471700 50.42514400 80.11428100

C 53.62977400 49.52692600 81.87303600

O 54.40280400 50.01757900 82.68280700

N 52.44007200 48.88259300 82.32048500

H 51.69440900 48.87127600 81.62383000

C 51.84833200 49.27262800 83.63252900

H 52.67949500 49.51570500 84.35718600

C 50.93660900 50.50266200 83.49266600

H 50.36943600 50.65796400 84.43386200

H 51.55694400 51.41319200 83.36694500

C 49.96757500 50.44058600 82.32822700

O 50.16834700 49.75147700 81.26794000

O 48.87762300 51.07054400 82.29468900

C 51.09183000 48.09303000 84.26713900

O 50.34504700 48.23725600 85.22242700

N 51.37116700 46.77017000 83.81317900

H 51.62397800 46.70019600 82.84586000

C 50.53441400 45.62912400 84.26186500

H 49.52997200 46.01946900 84.61633900

C 50.22803500 44.59233600 83.16628500

H 50.35162300 43.56917700 83.56359300

H 49.14965700 44.66443700 82.88819600

C 51.00896900 44.67109200 81.83901200

H 50.96416700 45.68969700 81.41339100

C 50.32682100 43.73564000 80.79050100

O 49.20928400 43.27426200 81.00493400

O 51.02752700 43.47631300 79.71415000

C 51.17166100 44.91478300 85.47166300

O 50.48384600 44.32173800 86.29185000

N 52.58207300 44.99111800 85.66821900

H 53.09311400 45.12066800 84.81910200

C 53.25488800 44.05862100 86.61285600

H 52.49131500 43.73401700 87.37144600

C 53.83317500 42.81924600 85.90713500

H 53.30482400 42.65786700 84.94197100

H 54.90162100 42.97599300 85.63030800

C 53.68884200 41.53473800 86.70777900

H 52.64480200 41.43578800 87.09403900

H 53.82512700 40.67392300 86.01846600

C 54.71438100 41.41037800 87.81754300

O 55.88259500 41.09024400 87.61740700

N 54.28121400 41.56015800 89.15078000

H 55.00049100 41.69168200 89.82567400

H 53.51641900 42.18822300 89.26141200

C 54.36057100 44.80813000 87.37997900

O 54.44671300 44.74029900 88.59761600

N 55.37536900 45.48280300 86.64965200

H 55.11960800 45.77722200 85.73078500

C 56.31410500 46.41746500 87.32358900

H 56.53298000 46.01354100 88.34458300

C 57.61422300 46.49241700 86.52678600

H 58.09005000 45.50541900 86.44588200

H 57.46041700 46.86959400 85.50560700

H 58.32943700 47.16370800 87.02034900

C 55.75576200 47.83689400 87.49201800

O 55.93674900 48.57193900 88.43747100

N 51.42942600 47.55449100 89.12657300

H 52.27635800 47.39652100 89.63202200

C 50.26397400 47.07336200 89.91818200

H 49.34021800 47.35350700 89.34909900

C 50.33791200 45.54054700 90.03552100

H 50.45315800 45.10337300 89.02156100

H 51.24608800 45.24195500 90.59953400

C 49.11654400 44.97060300 90.68136200

C 47.92593900 44.86118700 89.96152200

H 47.87861900 45.20204300 88.91407800

C 46.79363800 44.32213100 90.56100300

H 45.86304400 44.24349500 89.98850000

C 46.84134200 43.88840200 91.88146200

H 45.94690400 43.46932700 92.35387500

C 48.02756200 43.98894200 92.60033900

H 48.06982700 43.64598500 93.63912900

C 49.16272800 44.52637900 92.00408700

H 50.09580300 44.60501900 92.57469900

C 50.23237700 47.71058800 91.31302000

O 51.10553900 47.69152200 92.15894100

N 48.84323800 39.74808200 90.12912700

H 48.85742900 38.87774300 90.61793800

C 48.70121300 39.51385800 88.66690600

H 48.69086700 40.52262000 88.17882800

C 49.92176100 38.72422400 88.16627300

H 50.83706100 39.10788100 88.66452300

H 49.84710100 37.65768900 88.46083100

C 50.10286400 38.83909500 86.68715000

C 50.73362100 39.96324000 86.14886200

H 51.05914600 40.78064000 86.81346400

C 50.97578700 40.05945700 84.78511700

H 51.47679400 40.94657300 84.37471000

C 50.57179700 39.01163900 83.94998500

O 50.89357200 39.12995100 82.61998300

H 50.35164800 38.51220400 82.14216100

C 49.90877700 37.89221100 84.46983300

H 49.56321300 37.08528400 83.81267200

C 49.68263100 37.81434400 85.83762400

H 49.17044000 36.93758600 86.25281900

C 47.39916700 38.79545200 88.28927100

O 46.54115500 39.19319300 87.52742100

N 46.57893100 39.96687500 83.31034700

H 47.47670300 40.06257700 83.73749500

C 46.63724700 40.47307900 81.90185100

H 46.33157100 41.56239600 81.85020000

C 48.06886600 40.32801800 81.33256700

H 48.81258500 40.72023800 82.05726000

H 48.31947500 39.27137100 81.12334800

O 48.22371800 40.95956700 80.09435700

H 48.32641200 41.90041600 80.26790400

C 45.67323400 39.59965300 81.09562700

O 45.86476800 38.48247600 80.66032900

N 46.22035800 38.70590900 77.16302600

H 46.60414900 38.53637800 78.07000600

C 47.24138000 38.47171700 76.10968000

H 46.74379200 38.67384000 75.12587600

C 48.39237700 39.47015900 76.29794600

H 47.95901400 40.48232200 76.44065800

H 48.94959700 39.25270700 77.23894500

C 49.33530200 39.47817500 75.10044900

H 49.41624500 38.45478400 74.67719300

H 48.90770600 40.09457000 74.27986200

C 50.74429600 39.93677300 75.43328900

H 51.16293400 39.42648500 76.31326700

H 51.41508000 39.72674000 74.58785900

N 50.84258000 41.40435900 75.64728000

H 50.89799600 42.01904900 74.72978300

C 50.82286200 41.97907300 76.80934800

N 50.68301500 41.29365400 77.98645200

H 50.04317500 40.49487200 77.94930200

H 50.48772800 41.92887000 78.77844000

N 51.04094500 43.36849100 76.92074100

H 50.98069400 43.84140800 75.98800100

H 50.32058700 43.74419400 77.55131000

C 47.76313000 37.02751600 76.07102900

O 47.68903700 36.25471600 75.13763800

N 46.32232800 45.59336800 85.56019300

H 45.32898000 45.58370500 85.65873900

C 46.70925300 46.22135100 84.26954200

H 47.83590700 46.29323200 84.27828600

C 46.27168400 45.35069400 83.06676100

H 45.16395400 45.43407800 82.94443300

C 46.95763700 45.84510900 81.80056500

H 46.57122500 45.31464600 80.91220900

H 46.81958700 46.92442200 81.61788100

H 48.04601500 45.65509600 81.84914300

C 46.63464300 43.88577900 83.25979300

H 47.71850400 43.77071400 83.43752600

H 46.10395000 43.41789600 84.10592200

H 46.39053000 43.28197100 82.36503600

C 46.08728500 47.61725500 84.12084500

O 44.93413100 47.93640400 84.33924000

N 46.97667500 50.27537000 74.38799900

H 46.53118300 51.16720700 74.34393900

C 46.00154100 49.21866200 74.75979400

H 46.50471500 48.23084000 74.58123000

C 45.63067000 49.32181900 76.24695900

H 45.13737800 50.29096500 76.46222600

H 44.87398800 48.54144100 76.47182600

C 46.84043300 49.13965200 77.15261100

H 47.54204800 48.38790100 76.72386900

H 47.42091100 50.08394200 77.19954200

C 46.41810000 48.67891700 78.54140000

H 45.52107200 49.23194400 78.89312900

H 46.10889700 47.61026400 78.48464100

C 47.55036600 48.77994800 79.55472600

H 47.35337200 48.09485900 80.42192000

H 48.52090500 48.42807400 79.11128500

N 47.73197400 50.18209000 80.06746000

H 48.54358100 50.24242600 80.75078100

H 47.90666100 50.79594200 79.29377300

H 46.89483800 50.47773700 80.53471500

C 44.69745200 49.21496200 73.94991700

O 44.04707000 48.23976800 73.63450100

O 55.01559000 48.27682700 86.43098100

C 54.42277700 49.55590000 86.49974200

H 53.68822400 49.61586500 87.31726700

H 55.16919700 50.34938300 86.60633500

H 53.91761900 49.63016000 85.52715700

O 49.07321500 48.38258200 91.57421600

C 48.91475300 49.00094000 92.83308000

H 48.96108500 48.27559300 93.65234400

H 49.65919800 49.78800600 92.99436900

H 47.91308300 49.43161600 92.75982600

O 46.96848400 48.56525400 83.69345800

C 46.54421200 49.90785700 83.60087000

H 45.74219600 50.02425000 82.86322200

H 46.20948400 50.29805300 84.56685900

H 47.46804900 50.42334700 83.26265900

O 44.46298300 40.19412300 80.87279400

C 43.46568100 39.45829600 80.19584100

H 43.78548600 39.14723100 79.18846200

H 43.16037500 38.57597000 80.76867700

H 42.64390800 40.17520200 80.13051400

O 47.22595800 37.58613800 88.90382900

C 46.06296200 36.83774300 88.62033500

H 45.15406100 37.36949900 88.92119200

H 46.00195700 36.56969900 87.56015200

H 46.20511300 35.94475700 89.23430600

O 60.34758500 40.54320800 85.61665400

C 61.29797300 39.50950300 85.46670800

H 60.84279000 38.52162100 85.59171800

H 61.81136700 39.56385400 84.50122700

H 61.99510900 39.71567600 86.28319500

O 48.36716700 36.60617700 77.22303900

C 48.82035900 35.27076700 77.29985500

H 48.00413900 34.55278200 77.16584700

H 49.61217000 35.06986500 76.57016000

H 49.21469800 35.21486900 78.31739400

O 44.23663700 50.45223900 73.59267500

C 43.06260400 50.54111700 72.81372600

H 43.17146000 50.02999600 71.85125100

H 42.19211400 50.14494500 73.34676000

H 42.96185700 51.61939800 72.66572500

O 57.79923100 45.64106800 73.43852000

C 59.20631000 45.60048100 73.33545500

H 59.58758800 46.36655700 72.65174200

H 59.69160100 45.70255900 74.31202700

H 59.38167300 44.60268900 72.92496400

C 47.72190100 49.99815800 73.14546700

H 47.08767300 49.93503600 72.24838500

H 48.45260300 50.80281500 72.99438600

H 48.27483400 49.05162500 73.24044500

C 46.96339300 46.20664900 86.73710600

H 46.59294100 47.21460700 86.97851700

H 46.78757000 45.55581100 87.61059200

H 48.05245400 46.27061900 86.58837600

C 53.62545500 46.00381200 68.53240800

H 54.29272400 46.87689800 68.59518400

H 53.91232600 45.41227600 67.65338500

H 52.60094600 46.36819700 68.37871300

C 59.72179700 43.37837600 83.73323500

H 60.12627000 44.39732900 83.78615200

H 60.55950900 42.68021400 83.58171300

H 59.06142000 43.31691200 82.85294800

C 44.96060800 37.96627500 76.98229000

H 45.07380200 36.87170900 76.99637700

H 44.27899000 38.25506000 77.80051300

H 44.49397700 38.24919400 76.02951600

C 47.83063000 40.64892800 90.70441500

H 46.79859600 40.27538000 90.61882600

H 48.05762500 40.79321000 91.76906500

H 47.87719700 41.63335800 90.21496200

C 45.55787700 40.62415200 84.14414100

H 44.56542100 40.46175800 83.70413100

H 45.57447900 40.16100600 85.13965500

H 45.70397400 41.71577500 84.26405100

C 50.76081600 50.46856400 77.24852500

H 49.73867600 50.59467600 77.63706200

H 51.45491400 50.88823400 77.99846100

H 50.84745900 51.05558000 76.32558700

C 51.33022200 48.96090900 88.70101200

H 50.47248300 49.07946800 88.02486100

H 51.21762300 49.67555600 89.53062900

H 52.24361600 49.22581700 88.14237500

C 52.50593400 44.26873300 81.87054600

O 52.87341400 43.46825200 82.76713600

O 53.24251700 44.68243100 80.91188200

C 51.08264900 43.43361400 73.18060000

O 51.55841800 43.60040500 74.36406500

O 50.55504500 42.29848500 73.06164700

Ca 52.80411900 44.51421500 78.65532900

**γGlu42-Arg75-Ca^2+^**

Dyad with Ca^2+^ ion.

N -5.10543400 -0.12748300 -0.58044600

H -5.38555900 0.55443000 -1.28001000

C -3.64252900 -0.22838100 -0.58349900

H -3.26906400 -0.87145700 -1.38944000

C -3.05312000 1.18908200 -0.81309500

H -3.40386100 1.47105800 -1.81384100

H -3.52455500 1.88955700 -0.11699400

C -1.52995600 1.46005200 -0.77189800

H -1.41183600 2.50288000 -1.07876200

C -0.60596600 0.60796500 -1.65320500

O -0.93949200 -0.59829000 -1.89915600

O 0.51459600 1.11142600 -1.94134300

C -3.19753200 -0.96984700 0.69480700

O -2.50906700 -1.99788600 0.59325600

N 1.85877600 -1.70669700 -1.50543600

H 1.69909600 -0.75734900 -1.85950900

C 3.11673800 -1.78755100 -0.74516200

H 3.81309900 -2.48122200 -1.23560600

C 3.84666000 -0.42416200 -0.64402700

H 4.80955700 -0.57701900 -0.14834800

H 4.06924100 -0.12933000 -1.67639500

C 3.02472500 0.67824800 0.05336300

H 1.95868100 0.48389900 -0.07239700

H 3.20738100 0.67471600 1.13326700

C 3.27474100 2.09521700 -0.51343200

H 4.07557700 2.60231100 0.02992000

H 3.57453300 2.03195600 -1.56538000

N 2.04488800 2.88637700 -0.43416200

H 1.24592600 2.43834300 -0.91788300

C 1.86591900 4.03706800 0.21141700

N 2.92410000 4.82576500 0.50936900

H 3.76724900 4.78533300 -0.03999100

H 2.81818300 5.60086100 1.14414700

N 0.63392100 4.41758500 0.56128000

H -0.08416600 3.70080000 0.80553900

H 0.48497900 5.36403900 0.87700100

C 2.83757100 -2.39930000 0.63412200

O 1.71165100 -2.65421800 1.06680300

C -0.94199900 1.36552700 0.67292600

O -0.45314400 0.23729600 1.01894800

O -0.98223000 2.38735600 1.38691800

O 3.94085700 -2.63736800 1.31525300

C 3.80094400 -3.22199400 2.63836300

H 3.19586100 -2.57115300 3.27097100

H 3.33353700 -4.20474400 2.56128100

H 4.81618300 -3.30483600 3.01997900

O -3.56033300 -0.61610400 1.90694700

C -4.23379800 0.63091800 2.23362400

H -3.49291100 1.43288700 2.25328700

H -5.03045600 0.82262900 1.51683600

H -4.63165400 0.47276600 3.23495400

C -5.79364600 -1.39693300 -0.82928300

H -5.63473700 -2.08003400 0.01150700

H -6.86785600 -1.21143900 -0.90029200

H -5.46285300 -1.90770600 -1.74801300

C 1.77722200 -2.63937600 -2.64776300

H 2.61882900 -2.53589500 -3.34582000

H 1.75921100 -3.67570800 -2.29282800

H 0.85103300 -2.43348900 -3.18910900

Ca -0.27254700 -1.79964100 -0.01234300

**γGlu42-Arg75**

Dyad without Ca^2+^ ion.

N -2.80001700 -1.38037100 1.13251200

H -1.88469200 -1.27688400 0.68306800

C -3.77261100 -0.59795300 0.37315100

H -3.94402400 0.38010700 0.83637400

C -3.33896500 -0.33869800 -1.08982700

H -3.06812700 -1.28753500 -1.56312400

H -4.22400800 0.04159700 -1.61632200

C -2.17576300 0.64561700 -1.36768100

H -2.27046100 0.98364600 -2.40304400

C -0.71909800 0.03197400 -1.28486300

O -0.57699800 -1.04467800 -0.64024900

O 0.17532000 0.69729100 -1.85765300

C -5.17859700 -1.23633200 0.39282300

O -6.16273600 -0.58672600 0.68279200

N 2.20287100 -2.01222100 -0.14876400

H 1.32872800 -1.47772800 -0.19861500

C 3.34290800 -1.12966700 -0.05349300

H 3.54922300 -0.57459300 -0.98976900

C 3.17632800 -0.10546700 1.10102900

H 2.94771000 -0.67903500 2.00658400

H 4.14403000 0.38762900 1.26382600

C 2.09193200 0.96326300 0.88827400

H 1.12197000 0.49455200 0.70219200

H 1.99013900 1.54314500 1.81368600

C 2.39996600 1.92162700 -0.27379200

H 3.36154900 2.43147600 -0.10304000

H 2.50426300 1.34918800 -1.19574400

N 1.34925100 2.89399900 -0.55475100

H 0.60694100 2.49461100 -1.13471900

C 1.01175500 3.94931500 0.23375600

N 1.96067700 4.32648700 1.19212800

H 2.91921900 4.10259700 0.96068800

H 1.85783500 5.28227300 1.50211600

N -0.13191800 4.55526900 0.08798500

H -1.36698600 3.68156500 -0.63686400

H -0.23842800 5.32478300 0.74372900

C 4.62074600 -1.92314200 0.23037000

O 4.71751200 -3.01573900 0.74128300

C -2.10921900 1.89918300 -0.50643700

O -1.82164700 2.98793600 -1.22782800

O -2.20536700 1.90754900 0.71256900

O 5.71885200 -1.20188500 -0.15237400

C 6.97459300 -1.82437300 0.12835200

H 7.09554600 -2.00004200 1.20150300

H 7.05880300 -2.78524800 -0.38751400

H 7.73892700 -1.13333000 -0.23088100

O -5.37919000 -2.54954400 0.08941300

C -4.34188000 -3.41971700 -0.38925000

H -4.19852300 -3.28640000 -1.46767400

H -3.40768500 -3.23836500 0.14323800

H -4.70707000 -4.43498600 -0.20993100

C -2.74155300 -1.01821100 2.54251400

H -3.68446400 -1.27437500 3.04438200

H -1.94609300 -1.59407700 3.02804100

H -2.54921100 0.05378600 2.70812800

C 2.22786900 -2.88026000 -1.31901900

H 2.43060300 -2.34579300 -2.26686500

H 2.98330500 -3.66616800 -1.20161500

H 1.24849600 -3.35515800 -1.41467000

**γGlu47-Arg75-Ca^2+^**

Dyad with Ca^2+^ ion.

N -6.25824600 2.08670700 0.72799500

H -6.64380100 1.51713000 1.47696200

C -6.44121000 1.35878100 -0.52509200

H -6.54696100 2.10424100 -1.32406400

C -5.19927500 0.49733100 -0.90011000

H -5.53037600 -0.35685500 -1.49493200

H -4.53957700 1.09441800 -1.53273600

C -4.35908200 0.04369200 0.30241500

C -2.96792200 -0.41596600 -0.08873100

O -2.52588600 -0.26117500 -1.26952000

O -2.20763300 -0.95640100 0.81531000

C -7.72313900 0.51422700 -0.63815800

O -7.92231400 -0.27946400 -1.53204100

N 8.22698800 -0.75674400 1.47628000

H 7.52176800 -1.42434500 1.76973500

C 7.68729300 0.10876400 0.44866900

H 7.65104300 -0.36992500 -0.55277600

C 6.26704000 0.58453500 0.83411100

H 6.30824400 0.90786900 1.87927800

H 6.01188800 1.46353500 0.23353300

C 5.15600500 -0.47049500 0.67921500

H 5.43763900 -1.41789700 1.15497400

H 4.26226700 -0.12476200 1.21705800

C 4.79202100 -0.73653800 -0.78870500

H 4.45744500 0.18247500 -1.27892300

H 5.66498400 -1.07875600 -1.34939600

N 3.75373500 -1.76969200 -0.95929700

H 4.07838000 -2.72853500 -0.98692400

C 2.42587000 -1.59756300 -1.04151400

N 1.87992700 -0.26429700 -0.88525100

H 2.35249800 0.25490800 -0.14589900

H 2.00830100 0.27513900 -1.74343500

N 1.51401000 -2.50741000 -1.23834200

H 1.88709100 -3.43820400 -1.41134100

H -3.42157200 -1.43945400 1.95137000

C 8.58921300 1.34108000 0.25582900

O 9.52455700 1.65455100 0.94973600

H -4.21268900 0.94119500 0.93129400

C -5.10674600 -0.93404400 1.24378300

O -6.31268200 -1.01914300 1.22491900

O -4.37194700 -1.61598200 2.12947700

C -6.89206900 3.40712200 0.76858400

H -6.42062500 4.06265800 0.02844900

H -7.97434800 3.38786700 0.57541900

H -6.72789500 3.85084200 1.75468600

O -8.61541600 0.80508500 0.32475700

C -9.82749800 0.02749900 0.29349000

H -9.59540700 -1.02959300 0.43825400

H -10.34021500 0.15593400 -0.66215100

H -10.43859500 0.40292500 1.11339700

O 8.18333200 2.03867600 -0.83107000

C 8.95713600 3.22112300 -1.12563300

H 8.91248600 3.92496900 -0.29189500

H 10.00004200 2.95585100 -1.31027200

H 8.50679400 3.65234000 -2.01883300

C 9.45436200 -1.46536900 1.11480900

H 9.37790200 -2.04211600 0.17524700

H 10.27066200 -0.74794600 1.01481800

H 9.71505400 -2.15540400 1.92080800

Ca -0.53677000 -1.30673500 -0.74358600

**γGlu47-Arg75**

Dyad without Ca^2+^ ion.

N 6.67554400 -0.47121900 -1.26049800

H 6.66087400 -0.85230300 -0.31106500

C 5.84416300 0.72287400 -1.19546500

H 6.05829300 1.33193700 -2.08904600

C 4.33643100 0.36900900 -1.25596400

H 3.76184900 1.25877600 -0.99072700

H 4.10464600 0.12822800 -2.30154800

C 3.89919100 -0.82132100 -0.37583500

C 2.43027500 -1.12606200 -0.49614600

O 1.64478200 -0.05506400 -0.59571600

O 1.98793400 -2.27803400 -0.50518200

C 6.10790800 1.66450600 -0.00438500

O 5.35319900 2.55037900 0.34331300

N -7.53857700 -1.05947700 -0.11054000

H -6.93619700 -1.87370300 -0.16594800

C -6.70735100 0.12298300 0.04091500

H -6.30584300 0.23853600 1.06622800

C -5.51738000 0.09580700 -0.94887500

H -5.92804300 0.00778700 -1.96139900

H -5.01003300 1.06380600 -0.88815100

C -4.48650100 -1.02181000 -0.71889800

H -4.94300700 -2.01274400 -0.85215300

H -3.72289000 -0.94678400 -1.50086200

C -3.78182800 -0.97064800 0.65051800

H -3.38134500 0.03408000 0.82885900

H -4.49919400 -1.15435300 1.45856400

N -2.69591100 -1.92154800 0.80942800

H -2.98330700 -2.89119800 0.83694500

C -1.45581100 -1.74478500 0.18217900

N -1.06122100 -0.68005700 -0.43904800

H -1.78911900 0.02299000 -0.52345300

H 0.68265900 -0.32943800 -0.58637200

N -0.64412200 -2.84818400 0.26968100

H -0.70311600 -3.33493000 1.15518200

H 0.33479600 -2.66503400 -0.01837000

C -7.53388300 1.38809600 -0.21128000

O -8.64836200 1.44617700 -0.67899800

H 4.42510400 -1.71804000 -0.71769900

C 4.26121400 -0.66774100 1.16918300

O 5.49233900 -0.45204200 1.37961900

O 3.33604800 -0.80177300 1.98629500

C 8.03161500 -0.27938100 -1.74832900

H 8.00412700 0.07010100 -2.79039000

H 8.64356800 0.43324400 -1.17159900

H 8.55053100 -1.24577000 -1.74856400

O 7.33409600 1.48921700 0.55383000

C 7.45883200 2.03456500 1.87034100

H 6.76076400 1.50845000 2.52681900

H 7.24734700 3.10788600 1.87978200

H 8.49069400 1.84779900 2.17674100

O -6.83078600 2.48253400 0.17098700

C -7.48538900 3.74129400 -0.04504300

H -7.70542500 3.88565900 -1.10587200

H -8.42187800 3.79020600 0.51658500

H -6.78805800 4.50115000 0.30716100

C -8.52093000 -1.25264400 0.95157600

H -8.09382700 -1.23289600 1.97160600

H -9.28343200 -0.47283600 0.88583300

H -9.01785600 -2.21682200 0.80962200

**γGlu47-Arg75-Arg184-Ca^2+^**

Triad with Ca^2+^ ion.

N -11.25451300 0.47234600 0.28219200

H -12.18155800 0.68818000 0.63237300

C -10.45087300 1.70205800 0.28329000

H -10.88965300 2.34863000 1.05239900

C -8.98717900 1.47236000 0.70702700

H -8.48849300 2.44912000 0.72299700

H -9.00758900 1.09631600 1.73586300

C -8.17473700 0.50812900 -0.16683700

H -8.59114000 -0.50195000 -0.09070700

H -8.23260400 0.83605500 -1.21025800

C -6.70784700 0.47379700 0.26415500

H -6.28062600 1.48436800 0.20812000

H -6.62535200 0.12151700 1.29978900

N -5.94489300 -0.43574000 -0.60393100

H -6.38732100 -0.71273300 -1.46887000

C -4.65227600 -0.75400000 -0.44131800

N -3.95260900 -0.23769200 0.56902400

H -4.35614800 0.46199800 1.16944300

H -2.99683500 -0.51743200 0.78018300

N -4.05349500 -1.59325200 -1.30027800

H -4.61232900 -2.14974400 -1.92688500

H -3.07353500 -1.84686700 -1.14616800

C -10.53696300 2.51777000 -1.01881000

O -9.60213500 2.78507300 -1.74828300

C 0.78670200 -2.13915400 1.96551200

H 1.62341600 -1.70455700 2.52215700

H 1.00790200 -3.19978300 1.82835600

C 0.75941100 -1.43484900 0.58664500

H 0.48430500 -0.38104000 0.69000200

C 2.10643900 -1.52118100 -0.10220200

O 2.83730400 -2.55605400 0.06157700

O 2.36119700 -0.71164800 -1.07281700

C -0.91657700 -0.59376600 3.04312900

O -1.81545000 -0.05601900 2.41736000

N 8.42022200 1.80975100 1.72460100

H 7.97055900 2.71741700 1.63052300

C 9.12577300 1.50642900 0.49044000

H 9.82074300 0.68199500 0.69561700

C 8.11592200 1.00569700 -0.57586300

H 7.46113400 0.30835200 -0.04115400

H 7.49233600 1.85637500 -0.89538100

C 8.75056600 0.33931900 -1.80273100

H 9.41897300 1.04345400 -2.30589500

H 9.38654200 -0.49794000 -1.48814200

C 7.74348900 -0.16004200 -2.84267500

H 7.05900900 0.63179800 -3.16263700

H 8.26222100 -0.50347700 -3.74153100

N 6.92408200 -1.31370900 -2.39019800

H 7.38689900 -2.21339200 -2.41822500

C 5.69186300 -1.28293400 -1.90240000

N 5.02027000 -0.16329100 -1.67811800

H 5.49220600 0.72892900 -1.69566500

H 4.03493200 -0.21575800 -1.36237100

N 5.01294900 -2.48212000 -1.66796700

H 5.62369400 -3.29492100 -1.67812500

H 4.44711000 -2.45682700 -0.79071600

C 9.99807300 2.62839100 -0.10761300

O 10.75195800 2.45903600 -1.04520800

C -0.25798600 -2.02942700 -0.46551000

O -1.45873700 -1.78925300 -0.29619900

O 0.22757800 -2.70443300 -1.45076900

C 9.27640500 1.83569900 2.91690500

H 9.69157000 0.83712600 3.08771400

H 10.10827200 2.55245800 2.85971800

H 8.66418000 2.09149800 3.78515900

O 9.80378100 3.80853300 0.49809400

C 10.59493900 4.91216500 -0.00439900

H 11.65789100 4.68598400 0.09415600

H 10.36265700 5.09576600 -1.05514000

H 10.32215600 5.76850200 0.60954500

O -11.80337700 2.90050600 -1.25469200

C -12.01723300 3.67599300 -2.45323200

H -11.44870800 4.60725800 -2.40821500

H -11.70744900 3.10975000 -3.33402500

H -13.08636600 3.87962600 -2.48240100

C -11.39623200 -0.25605700 -0.98065100

H -12.03639700 -1.12461800 -0.80455700

H -11.84021900 0.33053400 -1.79966900

H -10.42765400 -0.63138300 -1.32187600

O -0.17820100 0.04072200 3.96138400

C -0.51038300 1.42392500 4.21890200

H -0.39742000 2.01959400 3.31051900

H -1.53864100 1.50313100 4.57661800

H 0.18797100 1.75291900 4.98625600

H -0.13251600 -2.38701400 3.86015300

C -0.46864800 -2.05735000 2.87033100

N -1.51629300 -2.95935500 2.44410100

H -1.90975400 -2.63661300 1.56642400

C -2.56761100 -3.17868200 3.43633900

H -2.13685200 -3.63664200 4.33284400

H -3.10372400 -2.26597100 3.74449200

H -3.29961200 -3.88134500 3.02967900

Ca 2.27051100 -2.77923300 -2.29563200

**γGlu47-Arg75-Arg184**

Triad without Ca^2+^ ion

N -4.94524500 -5.87327800 1.10496100

H -5.83686800 -6.22924500 1.43365000

C -5.05711100 -4.41726300 0.97047000

H -5.70350700 -4.10279800 1.80214600

C -3.73426500 -3.65154500 1.16507700

H -3.97725100 -2.58300500 1.13003200

H -3.38622900 -3.87078900 2.18041000

C -2.59939800 -3.92849900 0.16956500

H -2.20400300 -4.93834500 0.33543000

H -2.96838200 -3.87916700 -0.85945800

C -1.45834000 -2.91970500 0.33090600

H -1.81388900 -1.91986600 0.06304100

H -1.14838400 -2.88420600 1.39009000

N -0.34687500 -3.24870500 -0.55024400

H -0.10155500 -4.22688600 -0.60073200

C 0.68364600 -2.38576800 -0.87372700

N 0.55486400 -1.09976200 -0.40112300

H 0.27853400 -1.03533300 0.57027100

H 1.35665900 -0.49393300 -0.57819000

N 1.68078400 -2.71631000 -1.63913100

H 1.57270800 -3.64863200 -2.03149900

H 3.09336100 -1.98421200 -1.37928900

C -5.81049700 -3.92997200 -0.28207100

O -5.49273200 -2.99649500 -0.98780600

C 5.93030900 0.58324600 -0.47103800

H 6.30883400 1.54466300 -0.11961000

H 5.69058900 0.68776600 -1.53262800

C 4.64060900 0.31660900 0.33836700

H 4.87759600 0.34132200 1.40775700

C 3.64429400 1.48121700 0.10073800

O 4.09794100 2.64602400 0.24574300

O 2.44805000 1.18871600 -0.21535900

C 7.07953300 -1.08321800 1.02613700

O 7.52639300 -0.52094300 2.00444400

N -3.48325300 3.83481800 2.21520200

H -3.53956300 2.87615900 1.88024500

C -3.53246600 4.73708100 1.07510600

H -3.53163700 5.76626200 1.45340100

C -2.27817000 4.53629400 0.19336600

H -1.42533400 4.48616400 0.87755800

H -2.35831900 3.55542900 -0.29905700

C -2.06113400 5.63200600 -0.85582300

H -2.93088200 5.70499200 -1.51793500

H -1.98239700 6.60528900 -0.35393200

C -0.81749900 5.42259800 -1.73215400

H -0.86307700 4.46181700 -2.25444000

H -0.79333900 6.19120900 -2.51282700

N 0.45331700 5.48378600 -1.01155500

H 0.80691500 6.39978500 -0.78031200

C 1.26283200 4.43406800 -0.69944100

N 0.83393800 3.17961100 -0.79572700

H -0.15511000 3.00091200 -0.84821500

H 1.50028600 2.36807700 -0.56351300

N 2.50698300 4.67012000 -0.28500800

H 2.91707200 5.57144500 -0.47533200

H 3.15479600 3.84552600 -0.04105600

C -4.78821900 4.62767200 0.19500900

O -5.30087800 5.55059200 -0.40201200

C 3.90895400 -0.99409700 0.08424500

O 3.35633200 -1.61726900 0.97723800

O 3.87055600 -1.32777100 -1.20583000

C -4.53056800 4.06230700 3.20872800

H -4.41370700 5.06402600 3.63598100

H -5.55953400 3.97581000 2.82106800

H -4.41344000 3.34063700 4.02181300

O -5.22632800 3.35101400 0.11552200

C -6.36860700 3.13775700 -0.73448300

H -7.22361300 3.71489100 -0.37534600

H -6.14457900 3.44012300 -1.76009400

H -6.57685200 2.06956700 -0.68612700

O -6.93254200 -4.66047900 -0.48295000

C -7.73456500 -4.26192600 -1.60855400

H -8.08848800 -3.23562700 -1.48360600

H -7.15642900 -4.32446600 -2.53333100

H -8.57430600 -4.95578800 -1.63332500

C -4.55796900 -6.64352800 -0.07891000

H -4.58559600 -7.70565100 0.18138200

H -5.20861700 -6.49365200 -0.95504800

H -3.53344000 -6.40945700 -0.37329900

O 6.46948400 -2.28925000 1.06019200

C 6.20889300 -2.84578500 2.35995500

H 7.02515700 -2.61605000 3.04641800

H 5.26790300 -2.43592300 2.73505700

H 6.11078000 -3.92193800 2.21375200

H 8.02219800 0.05497000 -0.47026900

C 7.06735700 -0.47962400 -0.38814000

N 6.98356600 -1.40504700 -1.50433500

H 6.06690100 -1.84316100 -1.52423300

C 8.05231900 -2.38930700 -1.58491800

H 9.01553800 -1.87369800 -1.68786900

H 8.12564600 -3.07007000 -0.72066300

H 7.91048400 -2.99837700 -2.48348300

Optimized **γ**Glu (charge: -1).

C 1.897852000 2.322933000 0.098767000

H 1.522391000 2.034315000 1.082993000

H 1.259266000 3.106136000 -0.315709000

H 1.844259000 1.441309000 -0.544206000

C 3.362638000 2.730032000 0.239873000

O 4.183877000 1.964578000 0.725667000

N 3.655675000 3.969523000 -0.216341000

H 2.933999000 4.599561000 -0.543724000

C 4.972371000 4.589431000 -0.141497000

H 5.457882000 4.289541000 0.787904000

C 5.844471000 4.126244000 -1.337811000

H 5.922878000 3.038299000 -1.253934000

H 5.305602000 4.349461000 -2.262677000

C 7.267943000 4.727339000 -1.464192000

H 7.164839000 5.805915000 -1.604912000

C 7.865053000 4.196383000 -2.786054000

O 7.434096000 4.571758000 -3.857858000

O 8.832556000 3.303454000 -2.667745000

C 8.121217000 4.491211000 -0.179519000

O 9.005084000 3.591392000 -0.225595000

O 7.853954000 5.205257000 0.814556000

C 4.712358000 6.111858000 -0.124018000

O 3.642098000 6.570658000 -0.535110000

N 5.722587000 6.844536000 0.371780000

H 6.594013000 6.332576000 0.627034000

C 5.680089000 8.291938000 0.402287000

H 4.676318000 8.616146000 0.128492000

H 5.919175000 8.662043000 1.404308000

H 6.396786000 8.728180000 -0.303178000

H 9.032289000 3.247637000 -1.648824000

Optimized **γ**Glu (charge: -2).

C 1.856266000 2.399209000 0.221823000

H 1.421632000 2.239862000 1.212632000

H 1.287629000 3.170558000 -0.303786000

H 1.773632000 1.457607000 -0.328291000

C 3.346578000 2.733137000 0.390159000

O 4.073080000 1.955809000 1.005578000

N 3.720032000 3.882177000 -0.190868000

H 3.028312000 4.553537000 -0.523470000

C 5.032984000 4.531519000 -0.129861000

H 5.522057000 4.242960000 0.802173000

C 5.905466000 4.095808000 -1.338311000

H 6.060748000 3.015326000 -1.281376000

H 5.334779000 4.269856000 -2.255065000

C 7.276101000 4.781454000 -1.516223000

H 7.113628000 5.858039000 -1.606062000

C 8.010162000 4.357298000 -2.863017000

O 8.994901000 5.074832000 -3.147864000

O 7.508884000 3.405580000 -3.504242000

C 8.162574000 4.578158000 -0.285193000

O 8.977168000 3.688603000 -0.156765000

O 7.964861000 5.454967000 0.721933000

C 4.716295000 6.058216000 -0.098806000

O 3.576167000 6.432114000 -0.497774000

N 5.697153000 6.815306000 0.349506000

H 7.147793000 6.046688000 0.555638000

C 5.426440000 8.238589000 0.337352000

H 5.178708000 8.619520000 -0.667141000

H 4.571634000 8.514333000 0.979868000

H 6.310340000 8.778937000 0.697453000

Optimized **γ**Glu with calcium ion.

C 2.169264000 2.040064000 -0.400616000

H 1.844663000 1.579512000 0.536729000

H 1.416511000 2.765389000 -0.720478000

H 2.239481000 1.244018000 -1.146873000

C 3.552724000 2.642958000 -0.194841000

O 4.513879000 1.951528000 0.139569000

N 3.641154000 3.980931000 -0.404977000

H 2.842895000 4.548374000 -0.665304000

C 4.868131000 4.733904000 -0.208334000

H 5.340908000 4.406794000 0.721658000

C 5.841417000 4.469109000 -1.385072000

H 5.948853000 3.385713000 -1.460706000

H 5.361958000 4.814868000 -2.308501000

C 7.249469000 5.091339000 -1.301267000

H 7.206787000 6.181132000 -1.367291000

C 8.186790000 4.559496000 -2.375724000

O 9.285034000 5.187073000 -2.568681000

O 8.054004000 3.347822000 -2.765807000

C 7.989338000 4.772779000 0.068744000

O 7.608649000 5.372974000 1.076446000

O 8.950972000 3.902258000 0.054241000

C 4.445614000 6.212483000 -0.092902000

O 3.358857000 6.591592000 -0.541327000

N 5.347265000 7.016610000 0.509892000

H 6.208226000 6.581698000 0.855266000

C 5.119193000 8.437820000 0.689995000

H 5.067748000 8.697941000 1.753604000

H 5.920359000 9.026717000 0.229432000

H 4.170174000 8.691579000 0.216722000

Ca 10.089417000 3.191120000 -1.695504000

**Full reference 28**

Gaussian 09, Revision B.01, Frisch, M. J.; Trucks, G. W.; Schlegel, H. B.; Scuseria, G. E.; Robb, M. A.; Cheeseman, J. R.; Scalmani, G.; Barone, V.; Mennucci, B.; Petersson, G. A.; Nakatsuji, H.; Caricato, M.; Li, X.; Hratchian, H. P.; Izmaylov, A. F.; Bloino, J.; Zheng, G.; Sonnenberg, J. L.; Hada, M.; Ehara, M.; Toyota, K.; Fukuda, R.; Hasegawa, J.; Ishida, M.; Nakajima, T.; Honda, Y.; Kitao, O.; Nakai, H.; Vreven, T.; Montgomery, Jr., J. A.; Peralta, J. E.; Ogliaro, F.; Bearpark, M.; Heyd, J. J.; Brothers, E.; Kudin, K. N.; Staroverov, V. N.; Kobayashi, R.; Normand, J.; Raghavachari, K.; Rendell, A.; Burant, J. C.; Iyengar, S. S.; Tomasi, J.; Cossi, M.; Rega, N.; Millam, J. M.; Klene, M.; Knox, J. E.; Cross, J. B.; Bakken, V.; Adamo, C.; Jaramillo, J.; Gomperts, R.; Stratmann, R. E.; Yazyev, O.; Austin, A. J.; Cammi, R.; Pomelli, C.; Ochterski, J. W.; Martin, R. L.; Morokuma, K.; Zakrzewski, V. G.; Voth, G. A.; Salvador, P.; Dannenberg, J. J.; Dapprich, S.; Daniels, A. D.; Farkas, Ö.; Foresman, J. B.; Ortiz, J. V.; Cioslowski, J.; Fox, D. J. Gaussian, Inc., Wallingford CT, 2009.
